# Study on the Effect of Oleic Acid-Induced Lipogenic Differentiation of Skeletal Muscle Satellite Cells in Yanbian Cattle and Related Mechanisms

**DOI:** 10.3390/ani13233618

**Published:** 2023-11-23

**Authors:** Bin Sun, Jianfu Sun, Qiang Li, Ying Wang, Enze Wang, Huaina Jin, Huan Hua, Qiyun Jin, Xiangzi Li

**Affiliations:** Engineering Research Center of North-East Cold Region Beef Cattle Science & Technology Innovation, Ministry of Education, Yanbian University, Yanji 133002, China; a2653152414@163.com (B.S.); sunjianfu4379@163.com (J.S.); liqiang8589@ybu.edu.cn (Q.L.); 13180937133@163.com (Y.W.); wez19980804@163.com (E.W.); m18243627151@163.com (H.J.); hh390584731@163.com (H.H.); 15567668191@163.com (Q.J.)

**Keywords:** Yanbian cattle, bovine satellite cells, oleic acid, transcriptome sequencing, differentially expressed genes

## Abstract

**Simple Summary:**

Skeletal muscle satellite cells can be induced to differentiate into various cells under different conditions. The manipulation of adipogenesis during the stage critical for intramuscular adipocyte formation is expected to enhance marbling. However, oleic acid is a crucial player in regulating lipid metabolism and the formation of intramuscular marbling. In this study, the effects of different oleic acid concentrations on adipogenesis-induced differentiation of skeletal muscle satellite cells in vitro were selected. The results showed that oleic acid treatment could induce lipid droplet formation in the Yanbian bovine satellite cells. The expression of genes related to adipogenesis and fatty acid metabolism was upregulated, whereas that of genes related to muscle formation was downregulated. Oleic acid can induce Yanbian bovine star cells and promote cell lipid transdifferentiation.

**Abstract:**

Skeletal muscle satellite cells have the ability to differentiate into various cells under different conditions. This study aimed to investigate the effects of different concentrations of oleic acid (50, 100, and 200 µmol/L) on the process of lipogenic transdifferentiation in Yanbian bovine satellite cells, as well as its molecular regulatory mechanism. After inducing differentiation with oleic acid for 96 h, it was observed that the addition of oleic acid resulted in the formation of lipid droplets in the bovine satellite cells, and the triglyceride content showed a dose-dependent relationship with the concentration of OA. qPCR results demonstrated a significant downregulation of myogenesis-related factors (*Pax3* and *MyoD*) and upregulation of lipogenesis-related factors (*C/EBP-β* and *PPARγ*) (*p* < 0.05). Fatty acid metabolism-related factors, SCD and PLIN2, were also significantly upregulated (*p* < 0.05). These finding were consistent with the results obtained from Western blotting. Transcriptome sequencing analysis identified 278 differentially expressed genes between the control group and the groups treated with OA. KEGG enrichment analysis showed that differentially expressed genes were mainly concentrated in the adenosine monophosphate-activated protein kinase signaling pathway and fatty acid metabolic pathway. Our study presents that the OA induction of Yanbian bovine skeletal muscle satellite cells can promote cellular lipid transdifferentiation and reveals the potential genes and pathways related to OA induction of these satellite cells.

## 1. Introduction

In recent years, high-end marbled beef has been increasingly recognized by the public. The increase in intramuscular fat content results in beef with a snowflake-like appearance, enhancing the taste by making the beef tender and juicy. Intramuscular fat deposition (marbling) is a trait regulated by multiple factors, and genes play a decisive role in the composition distribution of the beef [1]. Therefore, the study of the molecular mechanisms of intramuscular fat deposition in beef has become a hot topic. Skeletal muscle satellite cells, as multipotent stem cells, can be induced to differentiate into various cells under different conditions. Studies have shown that skeletal muscle and adipose tissue development often have reciprocal relationships, where muscle satellite cells can be induced to transdifferentiate into mature adipocytes through the ectopic expression of adipogenic differentiation factors [2]. Oleic acid (C18:1 n-9, OA) is the most representative monounsaturated fatty acid in beef. OA is a critical regulator of lipid metabolism and intramuscular marbling formation. OA also regulates lipid metabolism, is integrated in the membrane structure, and is directly involved in the fatty acid composition of membrane phospholipids. It regulates the structural properties of phosphatidylethanolamine (PE) membranes differently than its counterparts trans-monoenoate (18:1 trans-∆9) and stearate (18:0) [3]. OA influences the transport and receptor activity of many enzymes, and the desaturation of essential fatty acids. OA can increase adipocyte intracellular fat content and fatty acid synthase activity and promote lipoprotein lipase expression [4,5,6]. Smith et al. reported [7] that intramuscular fat content is closely and positively correlated with OA content, but the exact action mechanism is unknown. that is, higher OA content is associated with higher intramuscular fat content in beef adipose tissue. Studies in our laboratory have found that OA promotes lipid droplet formation in skeletal muscle satellite cells and precursor adipocytes of Yanbian cattle. Therefore, we here used the established Yanbian bovine skeletal muscle satellite cells as a model to investigate the effect of OA on the lipogenic differentiation of these satellite cells. Meanwhile, lipogenic differentiation pathway-related genes in the satellite cells were screened and analyzed through bioinformatics by using transcriptome sequencing. These DEGs were annotated and enriched to provide a molecular theoretical basis for investigating the molecular regulation mechanism of the OA-induced lipogenic differentiation pathway in Yanbian bovine skeletal muscle satellite cells and to further elucidate the molecular mechanism of the *PLIN2* gene in lipogenesis.

## 2. Materials and Methods

### 2.1. Induction of Differentiation of Yanbian Bovine Skeletal Muscle Satellite Cells

This experiment used the Yanbian bovine skeletal muscle satellite cells that had been identified and preserved by the Engineering Research Center of the Ministry of Education for Science and Technology Innovation of Beef Cattle in the Northeast Cold Region of Yanbian University. The liquid-nitrogen-preserved cells were revived, inoculated in cell culture flasks, and incubated at 37 °C with 5% CO_2_ in an incubator. The cell basal culture medium was changed every 24 h. After the growth density of the cells reached 80%, they were digested with 0.25% trypsin and added to eight 6-well plates, divided into four groups. Complete culture medium was added to each well to continue the culture. When the density of the 6-well plates reached 90%, the culture medium was aspirated. The cells were washed twice with PBS containing 1% PS. The differentiation medium containing different OA concentrations, prepared in advance, was added to induce lipogenic differentiation. The cultures were washed twice with PBS containing 1% PS. A blank control group (CON, 2% HS), a 50 µM OA treatment group (OAL, 2% HS + 50 µM OA), a 100 µM OA treatment group (OAM, 2% HS + 100 µM OA), and a 200 µM OA treatment group (OAH, 2% HS + 200 µM OA) were established. The differentiation medium was changed every 48 h in two 6-well plates for each treatment. After 96 h of differentiation, the cells and culture medium were collected for subsequent experiments.

This experiment was initially designed to culture bovine satellite cells (BSCs) for 6 days. At 6 days, most BSCs were filled with lipid droplets after OA induction. However, the culture medium had to be changed very carefully and gently after 4 days. This is because most cells containing lipid droplets become fragile and the droplets easily break the cell membrane, come out of the cells, and float on the surface of the culture wells, thereby making it difficult to collect intact cells containing lipid droplets for oil red O staining. Therefore, we harvested the BSCs on day 4 for Oil Red O staining and the subsequent experiments.

### 2.2. Measurement Index and Method

Following the induction of differentiation of the satellite cells for 96 h, cell size and viability determination, oil red O staining, lipid droplet area analysis, triglyceride content determination, and the related gene expression assay were performed, and three replicates were established for each index to be measured.

### 2.3. Cell Size and Viability

The effect of OA on the satellite cells was evaluated by measuring cell size and viability by using the Taipan blue staining method. Oil red O staining and lipid droplet area analysis were performed to determine whether the cells were differentiated in the direction of lipogenesis. These two analyses were performed according to the instructions of the oil red O staining kit.

### 2.4. Triglyceride Concentration Measurement

The concentration of triacylglycerol (TAG) was measured using a TAG Quantitative Assay Kit (Applygen, Beijing, China) according to the manufacturer’s instructions. After 96 h of differentiation, the BSCs were washed with PBS to remove the medium, and the cells were lysed with Radio-ImmunoPrecipitation Assay lysis solution at ambient temperature for 10 min. Lipase decomposes TAG in cell lipid droplets to release glycerol, and the amount of glycerol released is measured through spectrophotometric detection at 570 nm.

### 2.5. Determination of Lipocalin (ADP) Content

ADP (Adiponectin) is an endogenous bioactive peptide or protein secreted by adipocytes. ADP content can be used as an index for identifying lipogenic differentiation. After 96 h of differentiation of the satellite cells, ADP content was determined using an Millibo bovine lipocalin enzyme-linked immunoassay kit and by referring to Li et al.’s method [8]. A standard curve was plotted to calculate the sample content, and GraphPad Prism 6.07 software was used for the analysis and for creating the graph.

### 2.6. Quantitative Testing of Related Myogenic Lipogenic Genes

After 96 h of the OA-induced differentiation of BSCs, relevant myogenic lipogenic genes were quantified through real-time fluorescence quantitative PCR to observe the effect of OA on lipogenic differentiation of the Yanbian cattle satellite cells at the molecular level. *GAPDH* was used as an internal reference gene. Table 1 presents the relevant specific primers used.

### 2.7. Protein Immunoblotting (Western Blotting)

After 96 h of OA-induced differentiation, the culture fluid was aspirated. The cells were rinsed three times with PBS pre-cooled to 4 °C in advance. An appropriate amount of protein-efficient lysis solution containing PMSF was added to each well, and the cells were lysed at 4 °C for 5 min. The cells were scraped. Lysates were collected and shaken at 4 °C for 30 min to fully release proteins. The proteins were extracted from the supernatant after centrifugation at 4 °C, 12,000 r/min, for 10 min. The protein concentration was determined according to Themor’s kit instructions. Finally, electrophoresis, membrane transfer, antibody incubation, and immunoreactivity steps in the Azure 600 multifunctional imaging system were followed to obtain photos and perform the analysis.

### 2.8. Transcriptomic Sequencing (RNA-Seq) Analysis

In this experiment, to determine the differentially expressed genes (DEGs) of the satellite cells involved in OA-induced lipid transdifferentiation, the skeletal muscle satellite cells were induced with different OA concentrations for 96 h. The DEGs of the transcriptome before and after OA induction were compared to those of the control group through RNA-seq analysis. This comparison helped to find *PLIN2* gene-related differentially expressed transcriptome information. This information will provide a theoretical basis for further analyzing the regulatory mechanism of the *PLIN2* gene and the effect of OA on lipid droplet formation in the skeletal muscle satellite cells of Yanbian yellow cattle. High-throughput sequencing was performed, and the obtained results were analyzed by Aimer Genetics. The main steps included in the technical process of the RNA-seq analysis were mRNA library construction and sequencing. Total RNA was isolated and purified using TRIzol reagent (Invitrogen, Carlsbad, CA, USA) following the manufacturer’s procedure. The RNA amount and purity of each sample was quantified using a NanoDrop ND-1000 (NanoDrop, Wilmington, DE, USA). The RNA integrity was assessed using an Bioanalyzer2100 (Agilent, Santa Clara, CA, USA) with RIN number >7.0 and confirmed by electrophoresis with denaturing agarose gel. Poly (A) RNA is purified from 1 μg total RNA using Dynabeads Oligo (dT)25-61005 (Thermo Fisher, Waltham, CA, USA) using two rounds of purification. Then, the poly(A) RNA was fragmented into small pieces using the MagnesiumRNA Fragmentation Module (NEB, cat.e6150, Ipswich, MA, USA) under 94 °C 5–7 min. Then, the cleaved RNA fragments were reverse-transcribed to create the cDNA by SuperScript™II Reverse Transcriptase (Invitrogen, cat.1896649, Waltham, CA, USA), which were next used to synthesise U-labeled stranded DNAs with *E. coli* DNA polymerase I (NEB, cat.m0209, Ipswich, MA, USA), RNase H (NEB, cat.m0297, Ipswich, MA, USA), and dUTP Solution (Thermo Fisher, cat.R0133, Waltham, CA, USA). An A-base is then added to the blunt ends of each strand, preparing them for ligation to the indexed adapters. Each adapter contains a T-base overhang for ligating the adapter to the A-tailed fragmented DNA. Single- or dual-index adapters are ligated to the fragments, and size selection was performed using AMPureXP beads. After the heat-labileUDG enzyme (NEB, cat.m0280, Ipswich, MA, USA) treatment of the U-labeled double-stranded DNAs, the ligated products are amplified with PCR by the following conditions: initial denaturation at 95 °C for 3 min, 8 cycles of denaturation at 98 °C for 15 s, annealing at 60 °C for 15 s, and extension at 72 °C for 30 s, and then final extension at 72 °C for 5 min. The average insert size for the final cDNA library was 300 ± 50 bp. At last, we performed the 2 × 150 bp paired-end sequencing (PE150) using an Illumina Hiseq2000 (San Diego, CA, USA) following the vendor’s recommended protocol.

### 2.9. Statistical Analysis

The data were expressed as mean ± standard deviation, and the fluorescence quantitative PCR data were calculated using Microsoft Excel 2010 software with a 2^−ΔΔCt^ mathematical model. One-way ANOVA and multiple comparisons were performed on the experimental data by using GraphPad Prism 6.07 software. *p* < 0.05 was considered statistically significant.

## 3. Results

### 3.1. Effect of Different OA Concentrations on the Size and Viability of Yanbian Cattle Skeletal Muscle Satellite Cells

**Figure 1:**
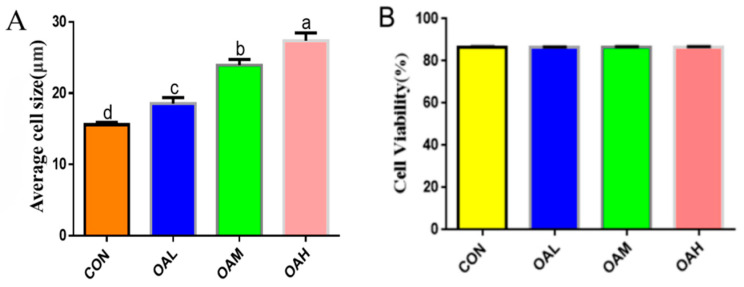
Effects of OA on the mean size (**A**) and cell viability (**B**) of BSC. Values with different superscript letters indicate significant difference (*p* < 0.05), whereas those with the same or no letter indicate that the difference is not significant (*p* > 0.05).

After 96 h of differentiation of the Yanbian skeletal muscle satellite cells induced by different OA concentrations, the effect of OA on the satellite cells was assessed by measuring the mean cell size and viability. The results revealed that the mean cell size was significantly increased in the OAL, OAM, and OAH groups (*p* < 0.05) compared to the CON group (Figure 1A), whereas no significant difference in cell viability (*p* > 0.05) was observed between the groups (Figure 1B).

### 3.2. Effect of Different OA Concentrations on Lipid Droplet Formation in Yanbian Cattle Skeletal Muscle Satellite Cells

**Figure 2:**
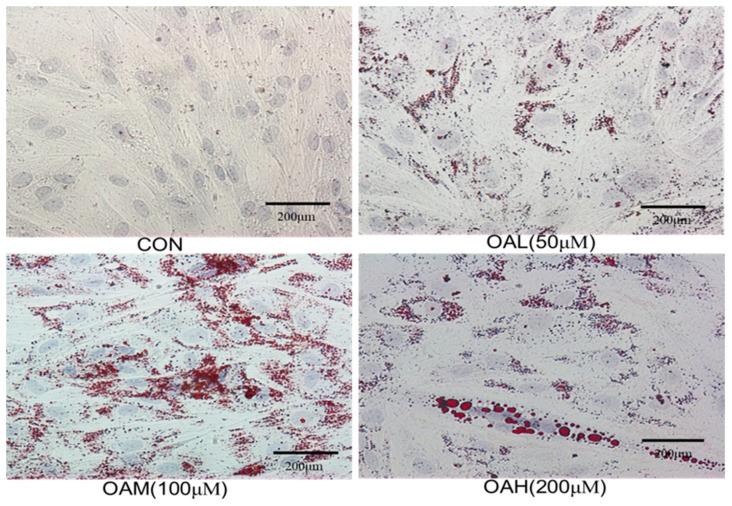
Oil red O staining of cells in each group (×20).

Bovine skeletal muscle satellite cells were treated with different concentrations of OA for 96 h, followed by Oil Red O staining of the treated and untreated cells. Intracellular lipid droplet generation is shown in Figure 2. The CON group exhibited obvious myotube formation, tight intercellular arrangement, and no lipid droplet generation (Figure 2). The induced group exhibited a large number of red lipid droplets around the nucleus, which became more obvious as the culture time was prolonged. Lipid droplet formation was accompanied by varying degrees of lipid droplet fusion.

### 3.3. Effect of Different OA Concentrations on Triglyceride Concentration and Lipid Droplet Area in Yanbian Cattle Skeletal Muscle Satellite Cells

**Figure 3:**
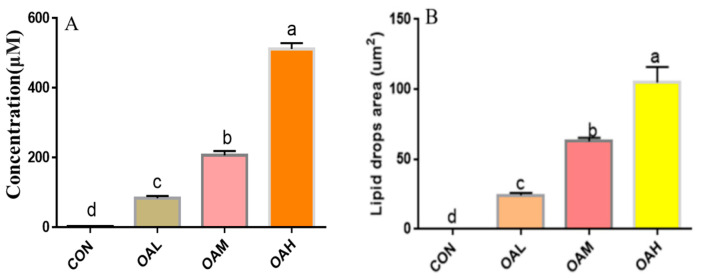
Effect of OA on triglyceride concentration (**A**) and lipid droplet area (**B**) of BSCs. Values with different superscript letters indicate significant difference (*p* < 0.05), whereas those with the same or no letter indicate that the difference is not significant (*p* > 0.05).

Following 96 h of induction of the satellite cells with different OA concentrations, the triglyceride concentration and lipid droplet area in the cells were examined to observe the effect of OA on the lipogenic capacity of the skeletal muscle satellite cells. The triglyceride concentration (Figure 3A) and lipid droplet area (Figure 3B) of the induction groups were significantly different from those of the CON group (*p <* 0.05). The triglyceride concentration and lipid droplet area were also significantly different among the treatment groups (*p* < 0.05). They exhibited a dose-dependent relationship with the OA concentration, and changes in the triglyceride concentration and lipid droplet area were consistent with those of changes in OA concentrations.

### 3.4. Effect of Different OA Concentrations on the ADP Content of Yanbian Cattle Skeletal Muscle Satellite Cells

**Figure 4:**
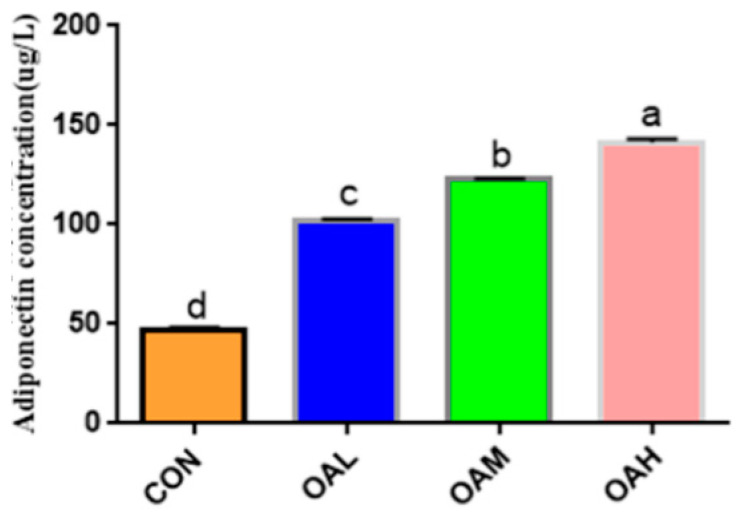
Effect of OA on the formation of bovine adiponectin. Values with different superscript letters indicate significant difference (*p* < 0.05), whereas those with the same or no letter indicate that the difference is not significant (*p* > 0.05).

After 96 h of induction of the satellite cells with different OA concentrations, the cell cultures were collected for ADP content determination (Figure 4). ADP was produced in all treatment groups. Compared to the CON group, the ADP content was significantly increased in the induction groups (*p* < 0.05), and the difference among the induction groups was significant (*p* < 0.05).

### 3.5. Effect of Different OA Concentrations on the Expression of Myogenic Lipogenic Genes in Yanbian Cattle Skeletal Muscle Satellite Cells

#### 3.5.1. Different OA Concentrations Downregulated the Expression of Myogenic-Related Genes

**Figure 5:**
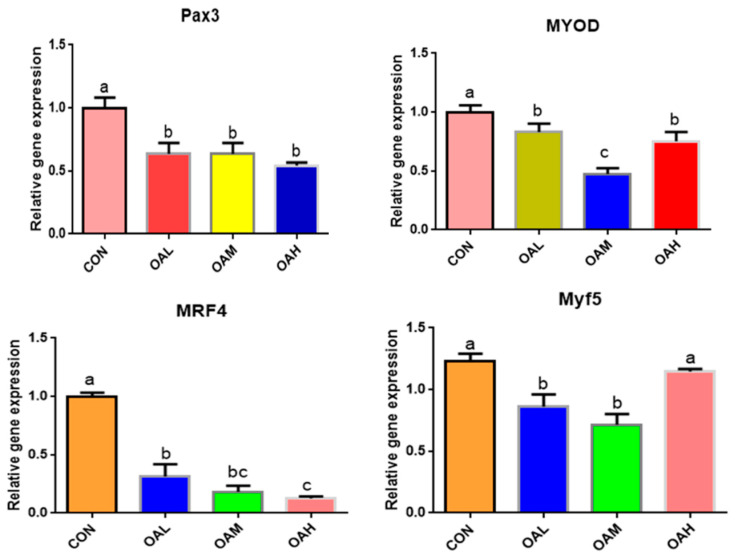
Effects of OA on the expression of myogenic-related genes. Values with different superscript letters indicate significant difference (*p* < 0.05), whereas those with the same or no letter indicate that the difference is not significant (*p* > 0.05).

After 96 h of OA induction, the expression of myogenic-related genes, *Pax3*, *MyoD*, *MRF4*, and *Myf5,* changed significantly (Figure 5). The expression of *Pax3*, *MyoD*, *MRF4*, and *Myf5* genes was significantly downregulated in the OA-treated groups compared to the CON group (*p* < 0.05). Differences between the OAL, OAM, and OAH groups in the *Pax3* gene were not significant (*p* > 0.05). Differences between the OAM group and the OAL and OAH groups in the *MyoD* gene were significant (*p* < *0.05)*, whereas those between the OAL and OAH groups were not significant (*p* > 0.05). Differences between the OAM group and the OAL and OAH groups in the *MRF4* gene were not significant (*p* > 0.05), whereas those between the OAL and OAH groups were significant (*p* < 0.05). The difference between the OAL, OAM, and CON groups and the OAH group in the *Myf5* gene was significant (*p <* 0.05), whereas that between the OAL and OAM groups and the CON and OAH groups was not significant (*p* > 0.05).

#### 3.5.2. The Expression of Lipogenesis-Related Genes Was Upregulated by Different OA Concentrations

**Figure 6:**
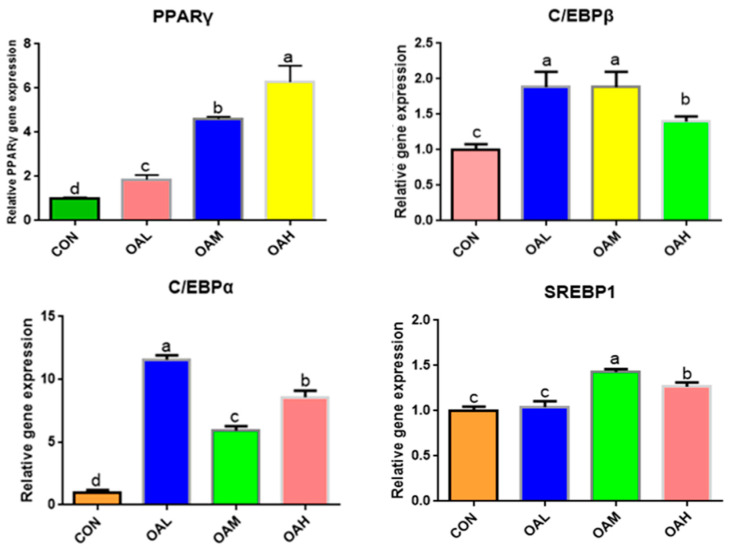
Effect of OA on the expression of adipogenesis-related genes. Values with different superscript letters indicate significant difference (*p* < 0.05), whereas those with the same or no letter indicate that the difference is not significant (*p* > 0.05).

The expression of adipose differentiation genes, such as *C/EBPα*, *C/EBPβ*, *PPARγ*, and *SREBP1*, changed significantly after 96 h of OA-induced differentiation of the skeletal muscle satellite cells (Figure 6). Compared to the CON group, *C/EBPα*, *C/EBPβ*, *PPARγ*, and *SREBP1* genes were significantly upregulated in the OA-treated groups (*p* < 0.05). The *PPARγ* gene was significantly different among the treatment groups (*p* < 0.05), exhibiting a measurement-dependent relationship. The *C/EBPβ* gene was significantly different between the OAH group and the OAL and OAM groups (*p <* 0.05), whereas the difference between the OAL and OAM groups was not significant (*p* > 0.05). The *C/EBPα* gene was significantly different among the treatment groups (*p* < 0.05). The *SREBP1* gene was significantly different among the treatment groups (*p < 0*.05) but not between the OAL and CON groups (*p > 0*.05).

#### 3.5.3. Expression of Fatty Acid-Associated Genes in Yanbian Cattle Skeletal Muscle Satellite Cells Treated with Different OA Concentrations

**Figure 7:**
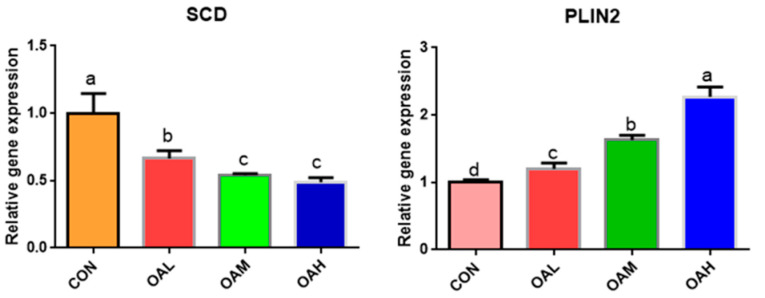
Effect of OA on fatty acid-related gene expression. Values with different superscript letters indicate significant difference (*p* < 0.05), whereas those with the same or no letter indicate that the difference is not significant (*p* > 0.05).

Among the fatty acid-related genes, the expression of *SCD* genes was significantly downregulated in the BSC cells after 96 h of OA induction (*p < 0*.05) in all OA-treated groups compared to the CON group (Figure 7). The differences were significant (*p <* 0.05) between the OAL group and the OAM and OAH groups, whereas those between the OAM and OAH groups were not significant (*p* > 0.05). The expression of *PLIN2* genes was significantly upregulated (*p* < 0.05) among all treatment groups compared to the CON group (*p* < 0.05) and exhibited a measurement-dependent relationship.

### 3.6. Effects of Different OA Concentrations on the Expression of Proteins Associated with Yanbian Cattle Skeletal Muscle Satellite Cells

**Figure 8:**
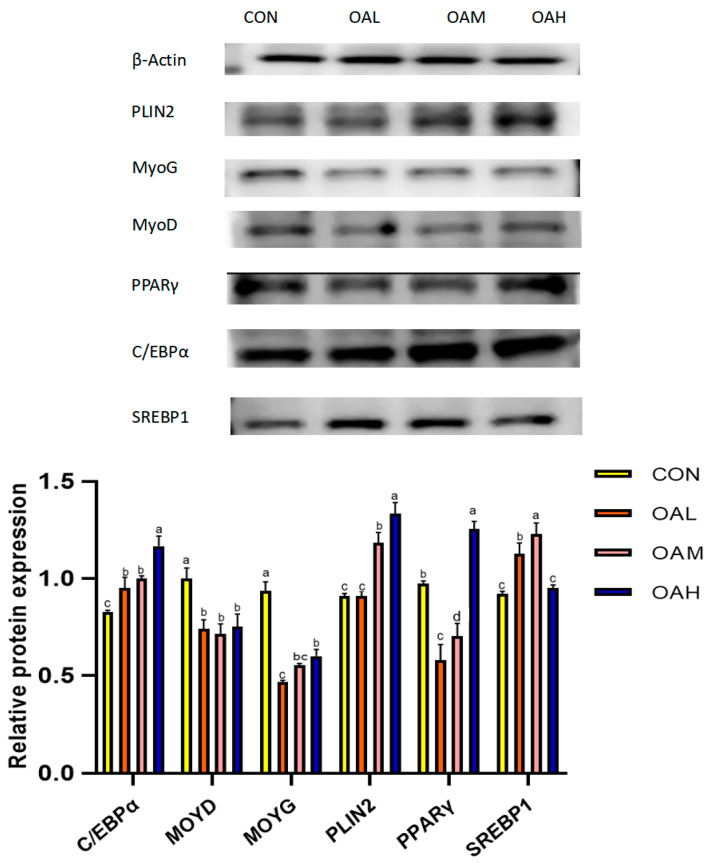
Effects of OA on protein expression. The original Western blot images are included in the Supplementary Materials. Values with different superscript letters indicate significant difference (*p* < 0.05), whereas those with the same or no letter indicate that the difference is not significant (*p* > 0.05).

Following 96 h of induction with different OA concentrations, the protein expression levels of myogenic and lipogenic genes and the internal reference gene *β-actin* were detected through Western blotting. The results revealed that the expression of *β-actin* in the CON, OAL, OAM, and OAH groups was basically maintained at the same levels (Figure 8), which proved that the initial protein addition was consistent. Compared to the CON group, the protein expression levels of the lipogenesis-related genes *PLIN2*, *C/EBPα* and *SREBP1* in the treatment groups exhibited an overall increasing trend, and those of the myogenesis-related genes, *MyoG* and *MyoD,* exhibited an overall decreasing trend. This was generally consistent with the trend of gene expression results.

### 3.7. Analysis of Differential Genes Involved in the Effects of Different OA Concentrations on Lipogenic Differentiation of Yanbian Cattle Skeletal Muscle Satellite Cells

**Figure 9:**
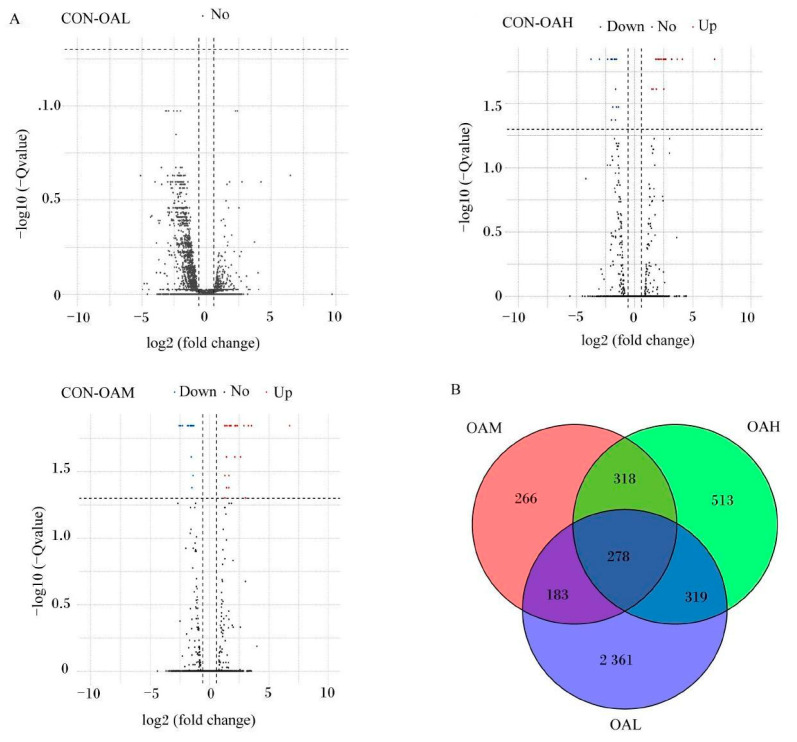
DEGs induced by different OA concentrations. (**A**) is a volcano map of DEG analysis. Red dots represent upregulated genes, blue dots represent downregulated genes, and gray dots represent genes that have not changed significantly. (**B**) is a Venn diagram of DEGs.

**Figure 10:**
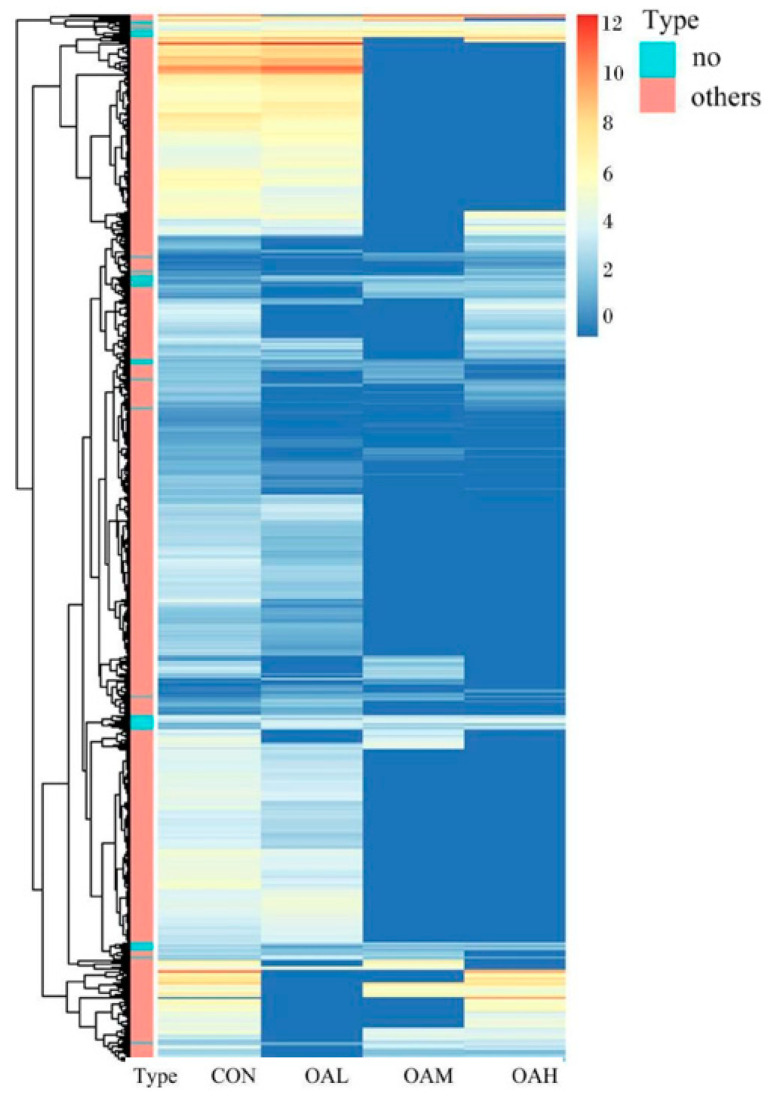
Clustering heat map analysis of DEGs in satellite cells with different OA concentrations. In the figure, the abscissa represents different experimental groups, and the ordinate represents the differentially expressed proteins of the group. The color blocks at different positions represent the relative protein expression levels at the corresponding positions. Red represents high expression levels, and blue represents low expression levels.

After 96 h of induction of the satellite cells by OA, results of the differential gene analysis (Figure 9A) revealed that, compared to the CON group, the OAL group had 3412 DEGs. Of them, 1168 genes were relatively upregulated, and 1973 genes were relatively downregulated. The OAM group had 1045 DEGs, of which 463 genes were relatively upregulated, and 582 genes were relatively downregulated. In the OAH group, 1428 DEGs were present, of which 704 genes were relatively upregulated, and 724 genes were relatively downregulated. As shown in the Wayne diagram, the combined analysis of these differential gene results revealed the presence of 278 DEGs in the satellite cells treated with different OA concentrations (Figure 9B). The clustering analysis revealed a clear pattern of differentially expressed gene grouping (Figure 10). 

### 3.8. Functional Analysis of DEG GO in Yanbian Bovine Skeletal Muscle Satellite Cells Treated with Different OA Concentrations

**Figure 11:**
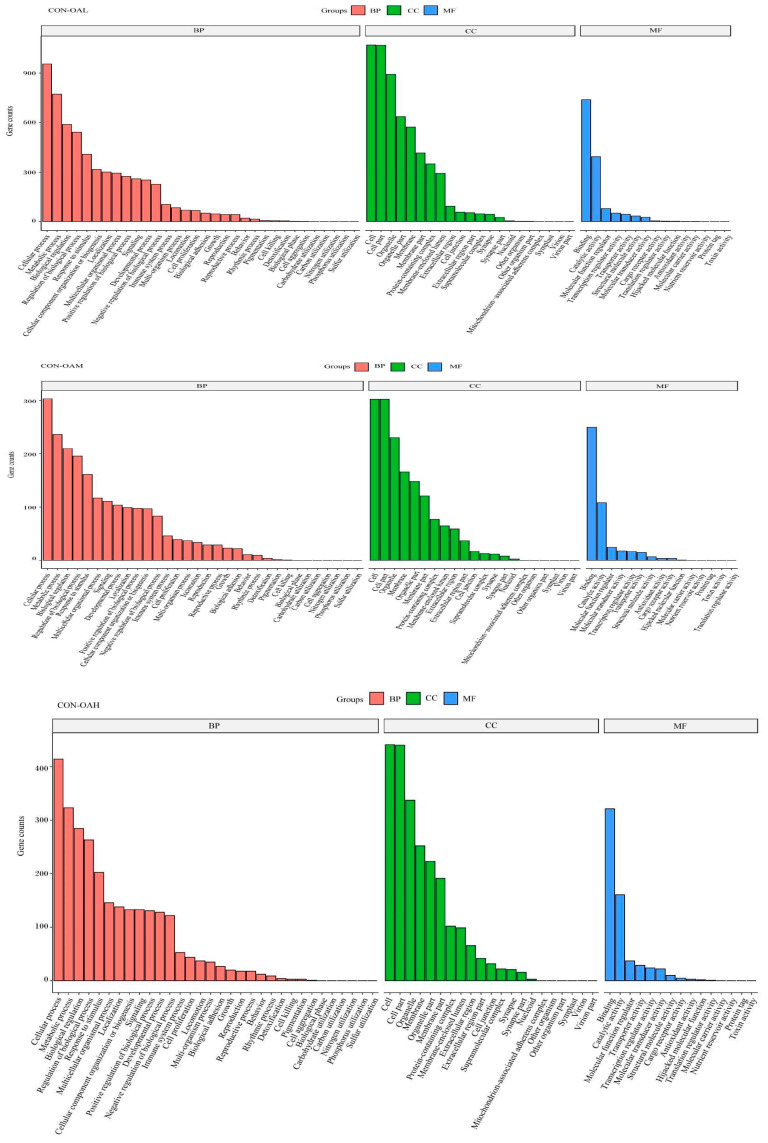
GO functional annotation classification of DEGs. The vertical axis represents the number of genes, and the horizontal axis represents the GO function annotation classification, from left to right.

To explore the biological functions of these DEGs, the GO enrichment results of DEGs at different OA concentrations were analyzed. The GO terms were categorized into molecular functions (MFs), biological processes (BPs), and cellular components (CCs). The top 10 GO entries with the smallest *p* values, that is, the most significant enrichment, in each GO category were selected for display (Figure 11). Compared to the CON group, the DEGs in the treatment groups were involved in various biological processes, including cellular processes, metabolic processes, and bioregulatory processes. In the molecular function category, most gene functions were associated with binding activity, catalytic activity, and molecular function regulatory activity. Most genes in the cellular component category were enriched in cells, cell parts, and organelles.

### 3.9. KEGG Enrichment Analysis of DEG-Associated Pathways in Yanbian Cattle Skeletal Muscle Satellites Treated with Different OA Concentrations

**Figure 12:**
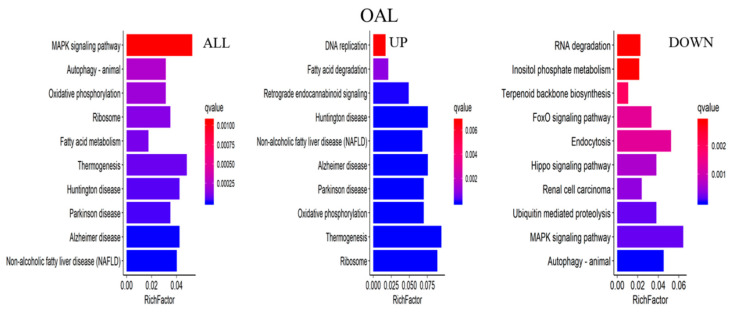

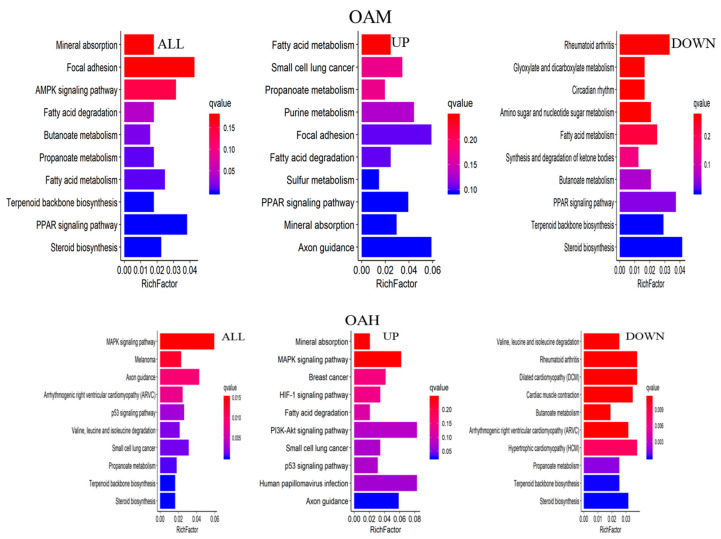
KEGG enrichment analysis of genes. The horizontal axis represents the enrichment coefficient, and the vertical axis represents the name of the pathway. The figure presents all the DEGs, the upregulated genes, and the top 10 signal pathways of the downregulated genes induced by different OA concentrations.

The major biochemical metabolic and signaling pathways associated with DEGs were explored through the KEGG enrichment analysis, and the top 10 enriched pathways were screened. Compared to the CON group, 1367 differential genes were annotated in the OAL group, which involved 86 pathways, and the most enriched pathways were the MAPK signaling pathway, thermogenic effect pathway, fatty acid metabolism pathway, etc. Among them, the 529 upregulated genes were mainly involved in the thermogenic effect pathway, ribosomal pathway, fatty acid degradation pathway, etc. The 838 downregulated genes were associated with the MAPK signaling pathway and cytokinesis pathway. In the OAM group, 444 genes were annotated, which involved 35 pathways, and the most enriched pathways were the PPAR signaling pathway, AMPK signaling pathway, fatty acid metabolism pathway, etc. Among them, the 203 upregulated genes were mainly involved in the PPAR signaling pathway, fatty acid degradation pathway, fatty acid metabolism pathway, etc. The 241 downregulated genes were mainly associated with the steroid biosynthesis pathway, PPAR signaling pathway, fatty acid metabolism pathway, etc. In the OAH group, 605 genes were annotated, which involved 57 pathways, and the most enriched pathways were the steroid biosynthesis pathway, MAPK signaling pathway, p53 signaling pathway, etc. Among them, the 289 upregulated genes were mainly associated with the p53 signaling pathway, fatty acid degradation pathway, MAPK signaling pathway, etc. The 316 downregulated genes were mainly involved in the steroid biosynthesis pathway, terpene skeleton biosynthesis, propionate metabolism pathway, etc. (Figure 12).

### 3.10. Analysis of the Signaling Pathway of the PLIN2 Gene in Yanbian Cattle

**Figure 13:**
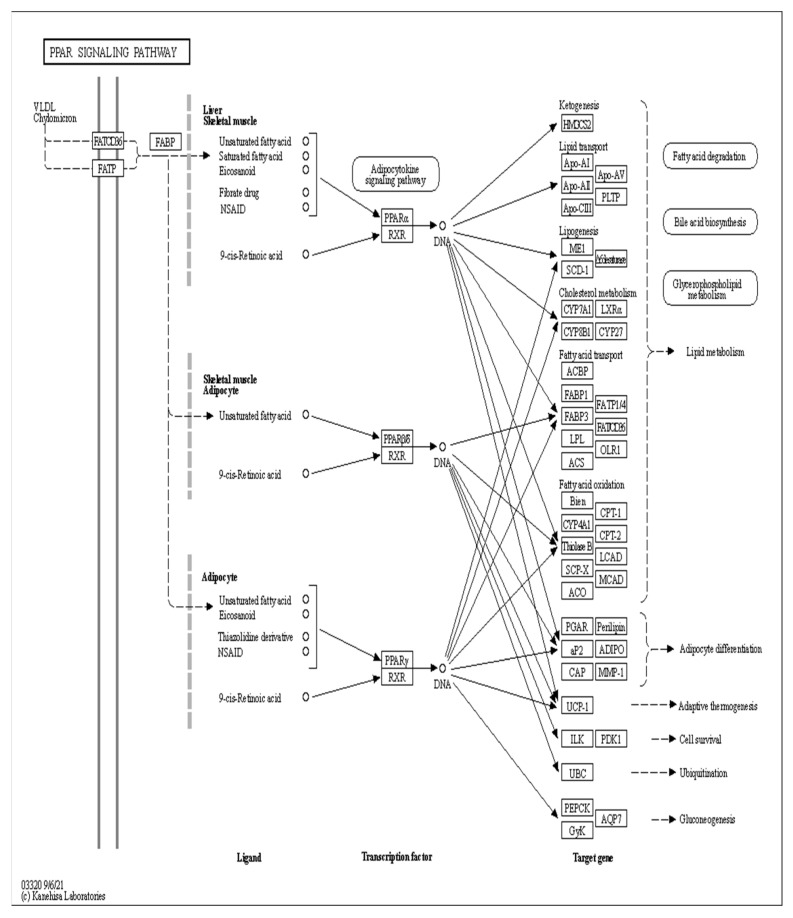
PPAR signaling pathway under the regulation of OA.

**Figure 14:**
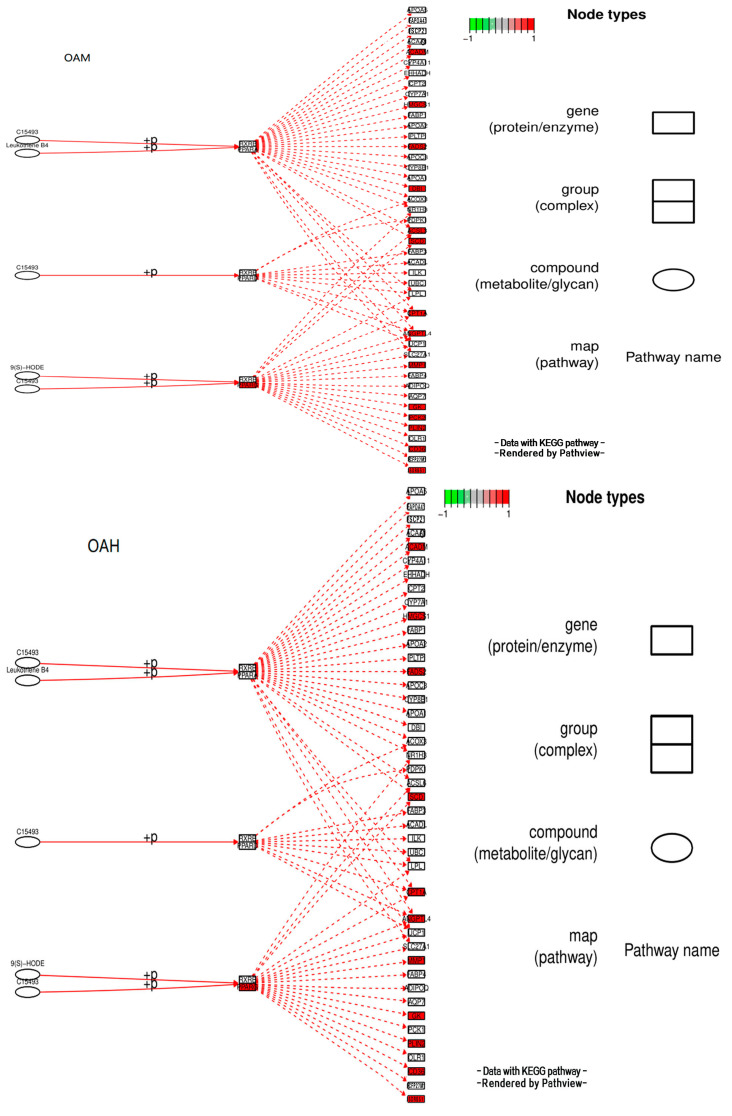
Pathview under the PPAR signaling pathway.

The signaling pathway of the *PLIN2* gene was analyzed on the basis of KEGG enrichment results of DEGs in the satellite cells induced with OA for 96 h (Figure 13 and Figure 14). PLIN2 is present only in the PPAR signaling pathway, and *PLIN2* gene expression is only regulated by PPARγ. This pathway is enriched in the liver, skeletal muscle, and adipocytes. When PPARγ is activated by binding to ligands, such as unsaturated fatty acids, eicosanoids, thiazolidine derivatives and NSAIDs, it forms PPAR/RXR heterodimeric transcription factors with the 9-cis-retinoic acid X receptor (RXR), which then binds to the PPAR initiation element (PPRE) upstream of the target gene promoter and finally regulates the transcription of these target genes. PPARγ plays a major regulatory role in adipose differentiation and lipogenesis in the PPAR signaling pathway. In addition, according to the pathway view (Figure 14), when the side of the added OA concentration changed, the target genes regulated by PPARγ also changed accordingly. However, matrix metalloproteinase (MMP)1, articulon protein 1 (SORBS1), PLIN2, and leukocyte differentiation antigen 36 (CD36) were all present in the pathway throughout the process, wherein MMP1 and SORBS1 were downregulated, whereas PLIN2 and CD36 were upregulated. This suggested a major molecular functional correlation among them and their crucial roles in lipogenesis.

### 3.11. Fluorescence Quantitative PCR Results of Effects of Different OA Concentrations on Differential Genes of Yanbian Bovine Skeletal Muscle Satellite Cells

**Figure 15:**
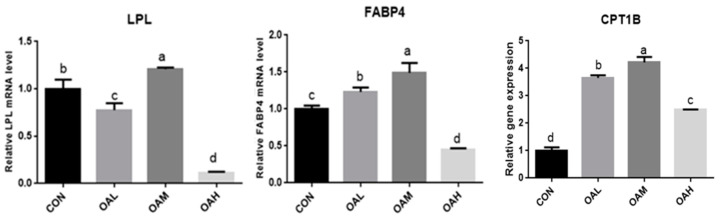
qRT-PCR verification of transcriptome sequencing results at different OA concentrations. Values with different superscript letters indicate significant difference (*p* < 0.05), whereas those with the same or no letter indicate that the difference is not significant (*p* > 0.05).

After OA induction for 96 h, the pathways enriched for DEGs changed with different OA concentrations. They were mainly enriched in MAPK, PPAR, AMPK, and other signaling pathways. The PPAR signaling pathway is a key player in adipogenesis and lipid metabolism. It is a key factor in regulating the body’s energy metabolism and fatty acid oxidation, while OA is a natural ligand of the PPAR system, combined with enrichment analysis and the main metabolic pathways related to adipocyte differentiation, To test the accuracy of transcriptome sequencing data, the candidate adipocyte differentiation-related genes *LPL*, *FABP4*, and *CPT1B* were screened from the PPAR metabolic pathways (Table 2). The transcriptome data were validated through fluorescence quantitative PCR (Figure 14). The correlation between the results of fluorescence quantitative PCR and transcriptome sequencing results was analyzed. The correlation was significant at the 0.05 level, and the gene expression trends were basically consistent. This indicated that the FPKM values obtained from the transcriptome were relatively accurate (Figure 15).

## 4. Discussion

Numerous studies have demonstrated that myosatellite cells are pluripotent stem cells capable of differentiating into various cell types. Li et al. [9] used OA to increase lipogenic gene expression in bovine muscle satellite cells in the absence of a PPARγ agonist. They revealed that OA can effectively increase lipogenic gene expression in BSCs in the absence of a synthetic PPARγ agonist. The present experiment showed that the morphology of the skeletal muscle satellite cells changed significantly after different OA concentrations were added, from spindle-shaped to rounded adipocytes, with a significant decrease in myotubes and intracellular lipid droplet production. This result is consistent with those of Li et al. [9]. In addition, no significant difference was observed in the cell viability assay, indicating that the currently used OA dosage is not toxic for the growth and differentiation of the skeletal muscle satellite cells. This provides a fundamental theoretical basis for future studies on fat deposition in meat. However, the optimum OA dosage to be used requires further investigation.

Muscle development are regulated by a series of signaling pathways, and normal muscle differentiation involves the expression of MRF family members, such as MYOD, MYOG, and PAX3/7 [10]. Pax3/7 is mainly located in the upstream regulatory region of the *MRF* gene promoter, plays a role in initiating *MRF* gene expression, and is crucial for embryonic skeletal muscle development [11]. MyoD is among the key members of MRFs, a transcription factor with a decisive role in skeletal muscle formation, differentiation, and maturation, and is a critical myogenic transcription factor in muscle regeneration. MyoD expression reflects the activation and differentiation of satellite cells. MyoD is a vital player in the differentiation of precursor cells to myogenic cells [12]. In this experiment, after 96 h of OA-induced differentiation of the satellite cells of Yanbian yellow cattle, it was shown that OA had a significant downregulating effect on *Pax3* and *MyoD*, indicating its ability to inhibit the differentiation of skeletal muscle satellite cells into myogenic cells. This result is consistent with those of Li et al. [9]. In their study, OA was used to increase lipogenic gene expression in bovine muscle satellite cells in the absence of a PPARγ agonist. They too found that OA significantly downregulated *Pax3* and *MyoD*.

PPARγ and C/EBPα are considered the most crucial regulators of adipogenesis [8]. PPARγ controls terminal adipocyte differentiation and is required for maintaining their differentiated state [13]. C/EBPα do not function effectively in the absence of PPARγ. In this experiment, OA significantly upregulated PPARγ and C/EBPα genes after 96 h of OA-induced differentiation of skeletal muscle satellite cells in Yanbian cattle. This indicated that OA could promote lipid deposition in the Yanbian cattle skeletal muscle satellite cells.

SCD is a rate-limiting enzyme that converts saturated fatty acids to monounsaturated fatty acids, leading to the formation of neutral lipid droplets [14]. Thus, SCD plays a key role in fatty acid metabolism. PLIN2 was originally referred to as an adipose differentiation-associated protein, which indicates its role in the early regulation of adipocyte differentiation [15]. PLIN2 surrounds the lipid droplet and assists the storage of neutral lipids within the lipid droplets [16]. During lipid droplet formation, PLIN2 is usually upregulated in parallel with stored lipids and appears on the surface of lipid droplets in the early stage of lipid droplet synthesis. In the present experiment, OA significantly upregulated the *PLIN2* gene and significantly downregulated the *SCD* gene after 96 h of the OA-induced differentiation of Yanbian cattle skeletal muscle satellite cells. This indicated that OA could promote fatty acid production in these satellite cells.

Adipose tissue, as a major endocrine organ, is a crucial player in regulating various physiological functions by secreting different hormones [17,18,19,20]. These adipose-derived hormones (also known as adipocytokines or adipokines) are secreted into the circulatory system where they act as important messengers between the adipose tissue and other tissues and organs [21]. Lipocalin is an adipokine that has received considerable research attention for its pleiotropic properties [22,23,24,25,26]. Lipocalin gene expression is tightly regulated by many transcription factors [27]. Upon binding to its receptors AdipoR1 and R2, lipocalin triggers a series of tissue-dependent signaling events [28,29], including AMPK and p38 MAPK phosphorylation and increased PPARα ligand activity [30,31,32]. This suggests that lipocalin production is closely related to AMPK, MAPK, and PPAR signaling pathways, as evidenced through the KEGG enrichment analysis of DEGs.

In this experiment, GO functional annotation and KEGG enrichment analysis of DEGs involved in OA-induced lipogenic transdifferentiation of Yanbian bovine skeletal muscle satellite cells revealed that the DEGs were mainly annotated to various biological processes and cell sites. The KEGG enrichment analysis revealed that the DEGs were mainly enriched in the AMPK and PPAR signaling pathways. The role of AMPK signaling pathway in regulating energy homeostasis is related to its effects on glucose and lipid metabolism, as well as on mitochondrial biogenesis and function [33]. The PPAR signaling pathway is mainly involved in regulating fatty acid metabolism, and cell proliferation and differentiation [34]. The functional annotation and enrichment analysis of KEGG–GO suggest that OA addition stimulates various metabolic pathways to promote the differentiation of the satellite cells toward lipogenesis, which may be because OA regulates the membrane structure of PE lipids, causing a negative membrane curvature strain, thus inducing a series of biological responses [35]. Cell membranes are mainly composed of phospholipids and proteins, and PE is the main phospholipid species in the plasma membrane [3]. PE is a lipid with a hexagonal HII phase preference [35]. Such hexagonal HII-phase-preference phospholipids can promote the fusion and fission of membrane bilayers [36,37], membrane permeability [38], protein transport [39], the regulation of chaperonin-like activity [3], etc. They are also involved in many cellular functions, such as endocytosis (membrane fission), extracellular (membrane–membrane fusion) processes, and the regulation of membrane protein activity [38]. Thus, the PE-conferred specific features are essential for membrane structure/function, and OA greatly promotes the tendency of the hexagonal HII phase [35], which is facilitated through the regulation of the negative membrane curvature strain. This regulation of the membrane structure partially explains the OA-mediated regulation of membrane and cellular functions, such as membrane fluidity, extracellular/endocytosis, cell division, signal transduction, and membrane protein activity, but the exact mechanisms need to be further explored.

**Figure 16:**
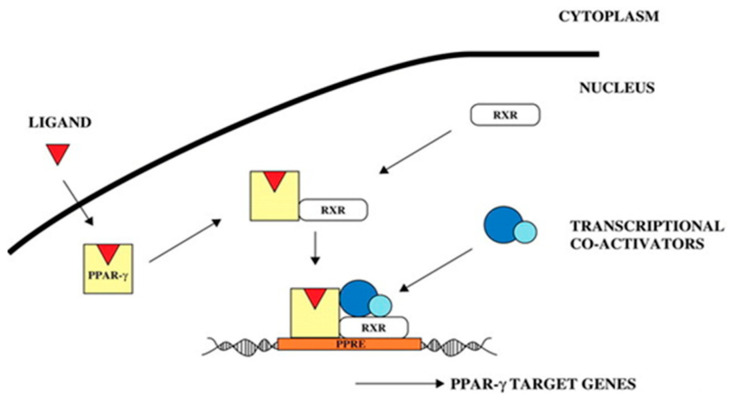
PPARγ signal transduction pathway [44].

PPARs play a chief role in lipid metabolism [40]. So far, three different PPAR isoforms, designated α, β (δ), and γ, have been identified. PPARγ is a key transcription factor of anabolism and plays an important role in lipid synthesis [41]. In the present study, PPARγ mRNA expression was observed in the skeletal muscle satellite cells after OA induction. The PPARγ expression level in the satellite cells increased significantly with increasing OA concentration and exhibited a dose-dependent relationship. Moreover, several studies have shown that PPARγ regulates *PLIN2* gene expression. To perform transcriptional functions, PPARs heterodimerize with RXR receptors and bind to their ligands, thus allowing for the transcription of their target genes (Figure 16), which have PPREs in their promoters, such as various lipid-metabolizing enzymes and proteins that constitute lipid droplets [42,43], while the *PLIN2* gene promoter region 5’-AGGTGAAA GGGCG-3’ sequence is a *PPARγ* PPRE [44]. This suggests that *PPARγ* controls *PLIN2* transcription through a functional PPRE located within the *PLIN2* promoter, as was also observed from the signaling pathway analysis of the *PLIN2* gene. In the presence of ligands, PPARγ regulates the expression of various lipid metabolism-specific genes by binding to PPREs in the promoter regions of downstream target genes such as *SCD*, *FABPs*, *LPL*, and lipid droplet-encapsulated protein (*PLIN*), thereby participating in the whole lipid metabolism process, such as lipid synthesis, transport, and deposition [45,46,47,48]. This is also consistent with the pathway view and the validation of adipocyte differentiation-related genes in the transcriptome data.

## 5. Conclusions

The study discovered that OA has the ability to promote the differentiation of Yanbian cattle skeletal muscle satellite cells towards lipogenesis. This promotion results in an increase in the generation of lipid droplets, the concentration of triglycerides, the content of lipocalin, and the expression of the *PLIN2* gene. Transcriptomic analysis revealed that the PLIN2 gene is involved in the PPAR signaling pathway and its expression is regulated by PPARγ. Additionally, there exists a significant molecular functional correlation between PLIN2, MMP1, SORBS1, and CD36, emphasizing the crucial role of PLIN2 in lipogenesis.

## Figures and Tables

**Table 1:** Primer information.

Genes	GenBank Accession No.	Primer Sequences (5′-3′)	Product Length in bp
*Pax3*	NM_001206818.2	GGCTGCGTCTCTAAGATCCT	158
		ATTTCCCAGCTGAACATGCC	
*MYF5*	NM_174116	CCCACCTCAAGTTGCTCTGA	115
		CCGTGGCATATACATTTGGTACA	
*MRF4*	NM_181811	TGGACCCCTTCAGCTACAGA	139
		ATGCTTGTCCCTCCTTCCTTG	
*PPARγ*	NM_181024	ATCTGCTGCAAGCCTTGGA	138
		TGGAGCAGCTTGGCAAAGA	
*C/EBPα*	GU947654	CCAGAAGAAGGTGGAGCAACTG	69
		TCGGGCAGCGTCTTGAAC	
*C/EBPβ*	NM_176788	CAACCTGGAGACGCAGCACAAG	143
		CGGAGGAGGCGAGCAGAGG	
*SREBP1*	NM_001113302	GCAGCCCATTCATCAGCCAGACC	146
		CGACACCACCAGCATCAACCACG	
*SCD*	NM_173959	TGCCCACCACAAGTTTTCAG	80
		GCCAACCCACGTGAGAGAAG	
*LPL*	NM_001075120	ACGATTATTGCTCAGCATGG	130
		ACTTTGTACAGGCACAACCG	
*FABP4*	NM_174314.2	AAACTTAGATGAAGGTGCTCTGG	134
		CATAAACTCTGGTGGCAGTGA	
*CPT1β*	NM_004377.4	ACACATCTACCTGTCCGTGATCA	72
		CCCCTGAGGATGCCATTCT	
*CD36*	NM_001278621.1	ACTGCGGATGGAATTTACAAAG	142
		ATGAGGCTGCATCTGTACCATTA	
*GAPDH*	NM_001034034.2	ACTCTGGCAAAGTGGATGTTGTC	199
		GCATCACCCCACTTGATGTTG	

**Table 2:** Differential gene enrichment of the PPAR signaling pathway.

Genes	ID	Signaling Pathways	Difference Multiplier log2(FC)	*p* Value	q Value	CON	OAL	OAM	OAH
*LPL*	bta03320	PPAR signaling pathway	−0.738 37	0.026 80	0.150245	5.29657	3.93057	6.40422	2.28923
*FABP4*			0.653 49	0.079 00	0.27557	28.9384	30.4699	79.478	26.6091
*CPT1B*			1.348 20	0.001 40	0.029393	1.79469	6.44781	4.92493	2.3348

## Data Availability

The datasets generated and/or analyzed during the conduct of the study are included in this published article. Upon reasonable request, the datasets of this study are available from the corresponding author.

## Supplementary Materials

The following supporting information can be downloaded at: https://www.mdpi.com/article/10.3390/ani13233618/s1, The original Western blot figures.
