# Supplementary material for: Study on the Effect of Oleic Acid-Induced Lipogenic Differentiation of Skeletal Muscle Satellite Cells in Yanbian Cattle and Related Mechanisms
Source: Animals (Basel). 2023 Nov 23;13(23):3618. doi: 10.3390/ani13233618 (PMC10705325; doi:10.3390/ani13233618)
Supplement: Supplementary file 1 [file animals-13-03618-s001.zip › animals-2602679-supplementary.pdf]

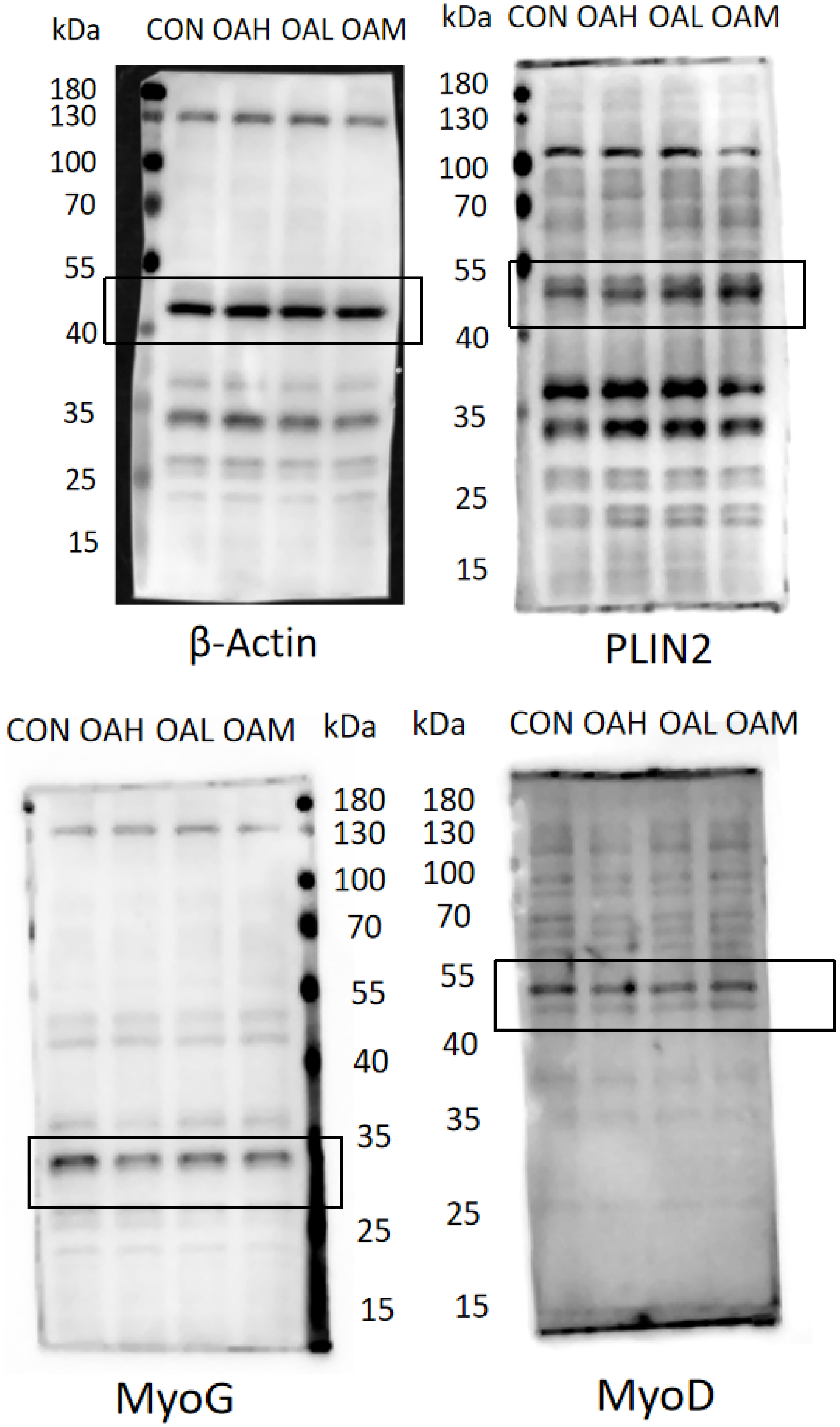
kDa
CON OAH OAL OAM
180
130
100
70
55
40
35
25
15
β-Actin
kDa
CON OAH OAL OAM
180
130
100
70
55
40
35
25
15
PLIN2
CON OAH OAL OAM
kDa
180
130
100
70
55
40
35
25
15
MyoG
kDa
CON OAH OAL OAM
180
130
100
70
55
40
35
25
15
MyoD

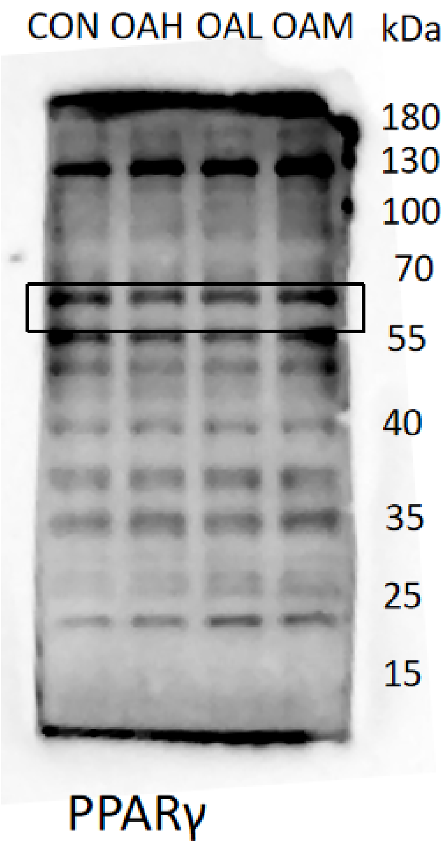

PPARγ

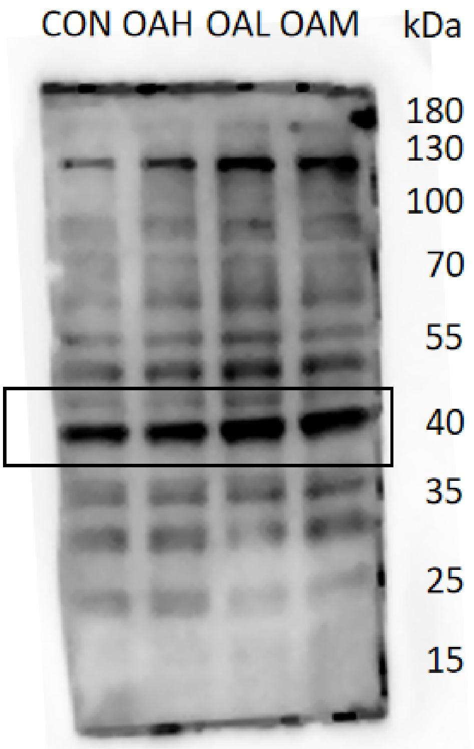

C/EBPα

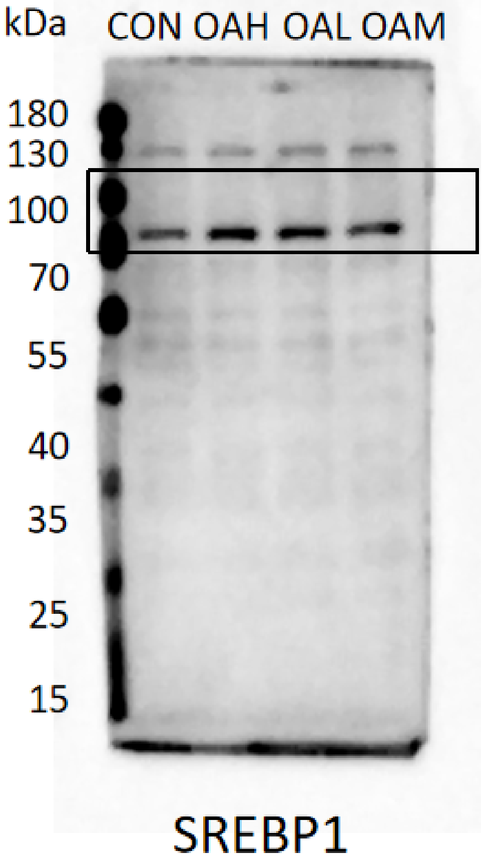

SREBP1
